# Response: Commentary: Bullous pemphigoid associated with COVID-19 vaccines: An Italian multicenter study

**DOI:** 10.3389/fmed.2023.1160672

**Published:** 2023-03-16

**Authors:** Anna Pira, Chiara Moltrasio, Damiano Abeni, Alberto Corrà, Angelo Valerio Marzano, Marzia Caproni, Giovanni Di Zenzo

**Affiliations:** ^1^Molecular and Cell Biology Laboratory, Istituto Dermopatico dell'immacolata (IDI)-IRCCS, Rome, Italy; ^2^Dermatology Unit, Fondazione IRCCS Ca' Granda Ospedale Maggiore Policlinico, Milan, Italy; ^3^Clinical Epidemiology Unit, Istituto Dermopatico dell'immacolata (IDI)-IRCCS, Rome, Italy; ^4^Section of Dermatology, Department of Health Sciences, University of Florence, Florence, Italy; ^5^Department of Pathophysiology and Transplantation, Università degli Studi di Milano, Milan, Italy; ^6^Rare Diseases Unit, Section of Dermatology, Department of Health Sciences, USL Toscana Centro European Reference Network-Skin Member, University of Florence, Florence, Italy

**Keywords:** COVID-19, SARS-CoV-2, autoimmune bullous disease, bullous pemphigoid, vaccine

We appreciate the insightful comments from Kasperkiewicz and Tukaj (1) regarding our study: “Bullous pemphigoid associated with COVID-19 vaccines: an Italian multicentre study”, recently published in Frontiers in Medicine (2).

We fully agree with the authors about the need to reconsider the relationship between COVID-19 vaccination and bullous pemphigoid (BP) onset, especially in the light of a recent work of Birabaharan and coworkers (3). In fact, the authors found no difference in risk of BP onset among two large cohorts of COVID-19 vaccinated individuals and unvaccinated matched ones, suggesting that the previously observed associations may be a random coincidence. Likewise, a recent study showed that circulating anti-SARS-CoV-2 antibodies do not cross-react with autoimmune blistering diseases (AIBDs) autoantigens, challenging the causal relationship between COVID-19 vaccination and AIBD new-onset (4).

However, we believe that other observations should be considered before drawing any conclusions.

## Not only molecular mimicry

Recently, several autoimmune diseases have been reported to be a consequence of COVID-19 vaccination, such as immune thrombotic thrombocytopenia (ITT), autoimmune liver diseases, Guillain–Barré syndrome, IgA nephropathy, rheumatoid arthritis, and systemic lupus erythematosus (5). In particular, vaccine-induced ITT was probably caused by platelet factor 4 (PF4) antibody-mediated activation through IgG-FcγR interactions, in addition to complement activation (6, 7). Surprisingly, vaccination-induced PF4 antibodies do not cross-react with the SARS-CoV-2 spike protein, suggesting a mechanism other than molecular mimicry underlying this event (8). In this context, it should be acknowledged that, in addition to molecular mimicry, there may be other mechanisms that could trigger AIBD onset following COVID-19 vaccination such as: (i) polyclonal activation due to adjuvant reaction, (ii) “bystander activation” of self-reactive lymphocytes; (iii) somatic mutation of immunoglobulin variable genes, and (iv) epitope spreading (9, 10). After COVID-19 vaccination, acute increase of type I interferons (IFN) expression, oxidative stress and DNA damage could induce both innate and adaptive immune responses (11). On the other hand, although methylated mRNA in COVID-19 vaccines is not immunogenic, some reports suggested the involvement of the pattern-recognition receptors (TLRs) 4, 7/8 and STING (12–14), especially in genetically predisposed patients (15). A potent adjuvant activity is due to the lipid nanoparticles, carrier vehicles that protect mRNA from degradation and allow intracellular delivery and endosomal escape, that could trigger inflammatory responses. In a mouse model, massive neutrophil infiltration and production of various inflammatory cytokines/chemokines including, among others, interleukin (IL)-1β/IL-6 and macrophage inflammatory protein-α and -β have been demonstrated (16). Thus, the cytokine milieu following COVID-19 vaccination could provide a causative link to BP induction in at least some of the reported cases.

## The AIBD onset following COVID-19 vaccination is a very rare event not easily measurable

Recently, Kasperkiewicz reported that in 932 post-SARS-CoV-2-vaccinal cases, only about 6% presented with new-onset AIBDs following COVID-19 vaccination, suggesting that this phenomenon is a very rare occurrence (17). Similar findings could be obtained considering that in 2021 only 30 Italian cases of new-onset AIBDs were reported (18). The only data on the Italian incidence of AIBDs was reported by Cozzani et al., on BP incidence in Liguria: ten BP cases per million per year were estimated (19), with a possible total incidence in Italy of 600 BP cases (10 x 60 millions of inhabitants) per year. Considering that most of the Italian population was vaccinated in 2021 and that BP is the most common AIBD, it could be estimated that < 5% of AIBD cases could occur following COVID-19 vaccination. As a result of this rare event, we think that the incidence rate estimation is not the ideal approach to assess the possible association between AIBD occurrence and COVID-19 vaccine. In fact, considering the data from Birabaharan et al., the rate of BP incidence per 100,000 person-years was 12 BP in the vaccinated cohort and 15 BP in the unvaccinated one, suggesting the risk of BP onset does not increase in vaccinated patients compared to non-vaccinated ones (3). The difference between 12 and 15 is not significant, but it should be noted that the data shown in Birabaharan et al. are compatible with a 57% excess in BP incidence due to vaccination (see the upper 95% confidence limit reported in the table 1 from Birabaharan et al.) (3). What would be expected in case of a causal association? Considering that 15 BP cases are not vaccination-dependent, if there was an actual causal relationship between BP onset and COVID-19 vaccination, we would expect a 6% increase of BP cases, corresponding to the frequency of this rare event. This would result in 15.9 cases per 100,000 person-years. Thus, to reach a statistically significant result (with α = 0.05 and β = 0.20), a sample size of over 2 billion individuals in each exposure group would be needed (20). In conclusion, the data from Birabaharan et al., that reported a not significant difference of BP onset between vaccinated and unvaccinated individuals, may suggest a random association with COVID-19 vaccination but cannot exclude that such association does exist.

## The real-life clinical observations are valuable

To date, the link between AIBD onset and COVID-19 vaccination is supported by many real-world clinical data. A recent review from Pira et al., reported 121 patients with new-onset AIBDs and 185 patients with relapse/worsening following SARS-CoV-2 vaccination (18). To find agreement between the real-life experience on patients with a new-onset/exacerbation of AIBD and other studies with conflicting results, vaccination could be considered as an anticipating factor that could induce autoimmunity, even without molecular mimicry, in genetically predisposed persons, by stimulating a pre-existent and sub-clinical autoreactivity against hemidesmosomal components. This phenomenon could slightly anticipate BP development without significantly modifying the disease incidence. On the other hand, as some studies reported, it is not unconceivable that COVID-19 vaccines may induce a suitable environment to make these subjects more prone to drug-induced BP, as reported in some case of dipeptidyl peptidase IV inhibitors users (21–23). Finally, although the meaning of the association between COVID-19 vaccines and AIBDs remains to be elucidated, we hope that Kasperkiewicz and Tukaj speculations, together with our observations, will be able to stimulate the scientific community to design additional investigations on this topic.

## Author contributions

AP contributed to the design and writing. CM, DA, AC, AM, and MC contributed to the writing. GD contributed to the design, writing, and supervision. All authors contributed to the article and approved the submitted version.
